# The relationship between home-based physical activity and general well-being among Chinese university students during the COVID-19 pandemic: the mediation effect of self-esteem

**DOI:** 10.1057/s41599-023-01717-8

**Published:** 2023-05-08

**Authors:** Mei Cao, Yongzhen Teng, Na Shao, Yijin Wu

**Affiliations:** 1 Tianjin Normal University, No. 393 Binshuixi Road Xiqing District, Tianjin, 300387 P.R. China; 2 Qufu Normal University, 80 Yantai North road, Rizhao, 266580, 276825 P.R. China

**Keywords:** Education, Sociology

## Abstract

This study aimed to investigate the mediating effect of self-esteem on the relationship between home-based physical activity and the general well-being of university students. A web-based questionnaire survey was conducted on 311 Chinese university students using the Physical Activity Rating Scale, Rosenberg Self-Esteem Scale, and General Well-Being Scale. The influence of home-based physical activity on self-esteem and general well-being in Chinese university students was explored using a one-way ANOVA analysis of variance. The mediating model was tested with regression analysis to determine the mediating effects of self-esteem between home-based physical activity and general well-being among Chinese university students during COVID-19. The amount of home-based physical activity had a significant effect on the general well-being (*F* = 3.46, *P* < 0.05) and self-esteem (*F* = 6.99, *P* < 0.01) of university students. The study found that self-esteem had a full mediation (*T* = 4.445, *P* < 0.001) between medium and large amounts of home-based physical activity and general well-being among university students, accounting for 32.5% of the total effect. The study concluded that self-esteem mediated the relationship between home-based physical activity and general well-being in university students during the COVID-19 pandemic. The findings in this study highlight the importance of home-based physical activity in increasing the general well-being of university students during the pandemic.

## Introduction

The COVID-19 epidemic had a significant impact on education worldwide, leading to the closure of schools in many countries and a shift toward online learning (Wathelet et al., 2020). For instance, in France, all levels of schools were shut down from March 17 to May 11 in 2020, necessitating students to remain at home and learn from online platforms. Similarly, in Singapore, the government mandated all students to study from home starting April 3, 2020, restricting their outdoor activities to only the purchase of basic daily necessities. In China, in the wake of the COVID-19 outbreak, all schools were compelled to shut down immediately, and teaching continued via online platforms (Wu et al., 2022).

Young adults between the ages of 16 and 24 are a crucial population to assess in light of their vulnerable stage of neurodevelopment (Kannarkat et al., 2020). In other words, school leavers and university students are particularly vulnerable to the negative effects of the isolation of COVID-19 (Wu et al., 2022). Specifically, they are susceptible to mental health (Cao et al., 2020), such as the increase in psychological stress, anxiety, and depressive disorder (Brooks et al., 2020; Marelli et al., 2021; Wathelet et al., 2020).

During the COVID-19 epidemic, university students could only do physical activity in their homes (Ammar et al., 2020; Papaspanos, 2021). Research has demonstrated that home-based physical activities have the potential to increase university students’ self-esteem (Yìğìter, 2014) and improve their general well-being (Maugeri et al., 2020). It is, therefore, because of these insights, necessary to further examine the influence of home-based physical activity on university students’ general well-being and investigate the mediating effects of self-esteem on the relationship between home-based physical activity and general well-being in university students during the COVID-19 epidemic.

### Physical activity and general well-being

Existing studies have indicated that there is a direct correlation between the level of physical activity and the general well-being of university students amidst the COVID-19 outbreak (Abdelbasset et al., 2021; Lukacs, 2021). Specifically, a higher level of physical activity leads to an enhanced level of general well-being in students. Hence, it can be inferred that engaging in home-based physical activities has a positive impact on the general well-being of university students during the COVID-19 pandemic.

### Physical activity and self-esteem

Physical activity has a significant impact on the level of self-esteem among university students. In a meta-analysis of more than 100 studies exploring the relationship between physical activity and self-esteem, Spence et al. (2005) found that participation in extracurricular activities in school had a consistently positive effect on the improvement of individual’s self-esteem (Yìğìter, 2014). Moreover, Hein and Hagger (2007) reported that active physical activity was associated with greater potential for improving self-esteem (Hein and Hagger, 2007). Given these findings, it is reasonable to believe that physical activity can have a beneficial effect on self-esteem among university students, particularly during the COVID-19 epidemic.

### Self-esteem and general well-being

Self-esteem is defined as people’s subjective evaluation of their self-worth (Rosenberg, 1979). Existing studies have reported that self-esteem had a direct relationship with one’s general wellbeing (Maslow and Lewis, 1987). It has been found that individuals with higher levels of self-esteem are more likely to have a higher level of general well-being (Sundaram and Patel, 2021).

**Fig. 1:**
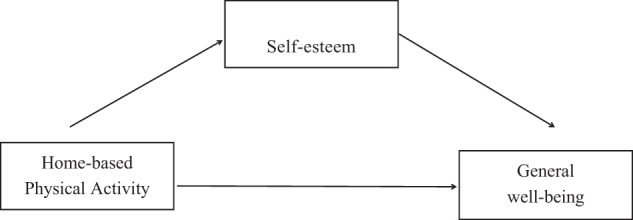
Conceptual framework for this study. Home-based physical activity as the independent variable, self-esteem as the mediator, and general well-being as the dependent variable.

This study aimed to investigate how home-based physical activity may affect general well-being among Chinese university students during the epidemic. Additionally, the study aims to explore the potential mediating effects of self-esteem on the relationships between physical activity and well-being. This study put forth the conceptual framework (in Fig. 1), and the following hypotheses were proposed:

Hypothesis 1: University students with higher levels of home-based physical activity exhibit higher levels of self-esteem and general well-being.

Hypothesis 2: The correlation between home-based physical activity and general well-being among university students can be mediated by self-esteem.

## Methods

### Participants

In this study, 350 university students from two universities in Sichuan and Shandong provinces were invited to fill in online questionnaires from April to May 2020. Of the 332 responses received, excluding those with incomplete or incorrect answers, 311 valid questionnaires were retained, reflecting a response rate of 93.7%. The retained questionnaires included responses from 101 freshmen, 88 sophomores, 79 juniors, and 43 seniors. The recovery rate was 94.85%, meaning that the majority of the targeted group participated. The participants in this study were ~20 years old (mean age of 20.02 ± 0.71) and came from various majors including environmental design, packaging engineering, financial management, business administration, journalism, law, network engineering, electrical engineering and automation, fine arts, translation, and business English.

### Instruments

Physical activity rating scale, Rosenberg self-esteem scale, and general well-being scale were used in this study. It takes about 8 min to fill in the three scales.

#### Physical activity rating scale

The physical activity rating scale has gained widespread usage for assessing the physical activity levels exhibited by university students. This scale has been thoroughly tested for both reliability and validity and employs a comprehensive five-point Likert scoring system. Within this scale, the amount of physical activity individuals engage in within their home environment is determined by the duration of their activities. Specifically, less than 20 min of home-based physical activities per day was regarded as a small amount of physical activity, 21–30 min is a medium amount of physical activity, and more than 31 min was a large amount of physical activity.

#### Rosenberg self-esteem scale

Rosenberg self-esteem scale is a widely used tool in the field of self-esteem research. It was originally developed by Rosenberg (1979) to measure both positive and negative feelings individuals have about themselves. This scale includes 10 items and utilizes a four-point Likert scoring method, whereby a score of 1 signifies “extremely inconsistent”, 2 denotes “inconsistent”, 3 corresponds to “consistent”, and 4 indicates “extremely consistent”. Half of the items were scored in reverse, and the higher the score is, the higher the level of self-esteem reaches. The internal consistency coefficient for this survey is 0.89, signifying strong reliability.

#### General well-being scale

General well-being scale has been consistently utilized in evaluating the general well-being of individuals. The scale strives to capture the subjective perception of one’s inner state by examining various dimensions of emotional and mental well-being. The National Center for Statistics of the United States originally developed the general well-being scale (GWB), which was revised by Duan in 1996 (Duan, 1996). The initial 18 items and six distinct dimensions were retained. These dimensions include an individual’s satisfaction and engagement in daily life, concerns about health, energy levels, emotional state, control over behavior, and relaxation. Compared to other tools measuring anxiety and depression, the General Well-Being scale has proven significantly efficient in evaluating an individual’s overall mental health, making it a valuable instrument in research. The scale operates on a scoring system where a higher score results in a higher level of well-being. The internal consistency coefficient of this survey is 0.85.

#### Statistical analysis

The data were analyzed using version 19.0 of Statistical Package for the Social Sciences (SPSS). To ensure the assumptions of one-way analysis of variance (ANOVA) were met, we first assessed the normality of the distribution. ANOVA was used to explore the influence of home-based physical activity on self-esteem and general well-being where physical activity was the independent variable and self-esteem and general well-being were the dependent variables. Additionally, we conducted a correlation analysis between the amount of physical activity, general well-being, and self-esteem. Finally, a mediation model was explored using regression analysis in SPSS to assess the mediation effect of self-esteem on physical activity and general well-being. Specifically, the following steps were taken: (1) general well-being was the dependent variable and medium to large amounts of physical activity served as the independent variable, (2) self-esteem was the dependent variable and medium to large amounts of physical activity were the independent variable, and (3) general well-being was the dependent variable, while medium to large amounts of physical activity and self-esteem served as independent variables.

## Results

### Variance analysis of the influence of the amount of physical activity on general well-being and self-esteem

The participants’ demographic characteristics are presented in Table 1. The homogeneity of variances was confirmed by the chi-square test (Levene’s statistic = 0.798, df1 = 2, df2 = 308, *p* = 0.455). One-way analysis of variance (Table 2) was performed using the amount of physical activity as the independent variable and general well-being as the dependent variable. The results showed that the amount of home-based physical activity had a significant effect on the general well-being of university students (*F* = 3.46, *P* < 0.05). Further analysis revealed that students who engaged in a small amount of physical activity had a lower level of general well-being, while those who engaged in a medium or large amount of physical activity exhibited higher levels of general well-being.

**Table 1:** Demographic characteristics of participants.

Demographic variables		*n*
Grade	Grade 1	101
	Grade 2	88
	Grade 3	79
	Grade 4	43
Sex	Male	41
	Female	270
Age	17	2
	18	30
	19	78
	20	94
	21	69
	22	26
	23	8
	24	3
Specialties	Liberal arts	287
	Science	24
Location	South	108
	North	203

**Table 2:** Comparison of the differences in the amounts of physical activity on general well-being and self-esteem (*x* ± *s*).

Amounts of physical activity	*n*	Self-esteem	General well-being
small	62	27.47 ± 4.78	75.09 ± 5.50
medium	106	29.70 ± 5.18	77.12 ± 5.29
large	143	30.11 ± 4.34	77.23 ± 5.88
*F*-value		6.99	3.46
*P*-value		<0.01	<0.05

To test for variance homogeneity, a chi-square test was performed with Levene’s statistic = 2.198, df1 = 2, df2 = 308, and *p* = 0.113. One-way analysis of variance (Table 2) was carried out, with the amount of physical activity serving as the independent variable and self-esteem as the dependent variable. The findings indicated that the amount of physical activity had a significant impact on self-esteem (*F* = 6.99, *P* < 0.01). Specifically, students with a small amount of home-based physical activity exhibited lower levels of self-esteem. Conversely, students with medium or large amounts of home-based physical activity displayed higher self-esteem.

### Correlation analysis of physical activity amounts, general well-being, and self-esteem

The findings indicate a positive association between the amount of physical activity engaged in by students and their self-esteem and general well-being, as evidenced by the data presented in Table 3.

**Table 3:** Correlation analysis between the amount of physical activity, self-esteem, and general well-being.

Variables	1	2	3
1 Amounts of physical activity	1	–	–
2 Self-esteem	0.190**	1	–
3 General well-being	0.126*	0.269**	1

*p < 0.05, **p < 0.01.

### Mediating effect of self-esteem between physical activity and general well-being

To test if self-esteem serves as a mediator between home-based physical activity and general well-being, an intermediary effect test was used to examine the mediating effect of self-esteem. General well-being and self-esteem scores were zero-centered, and home-based physical activity scores were virtually coded. Regression analysis was then performed to assess the mediating effect.

Specifically, the mediating effect was evaluated using general well-being as the dependent variable (*Y*), self-esteem as the mediating variable (*M*), and medium (*X*_1_) and the large amount of physical activity (*X*_2_) as the independent variables.

The findings from Table 4 demonstrate that medium or large amounts of home-based physical activity are significantly associated with general well-being and self-esteem. However, when self-esteem is considered as a mediating variable, the medium amount of home-based physical activity did not significantly predict general well-being (*T*_1_ = 1.556, *P* > 0.05) and a large amount of home-based physical activity also did not significantly predict general well-being (*T*_2_ = 1.617, *P* > 0.05). Meanwhile, self-esteem remained a significant positive predictor of general well-being, indicating that self-esteem fully mediated the relationship between medium and large amount of home-based physical activity and general well-being (*T* = 4.445, *P* < 0.001). The results indicate that the mediating effect accounted for 32.5% of the total effect.

**Table 4:** Mediation analysis of self-esteem between physical activity and general well-being.

Steps	Regression equation	*t*-value	*p*-value
Step 1	*Y* = 0.152*X*_1_ + 0.235*X*_2_	2.023^*^	0.044
		3.140^**^	0.002
Step 2	*M* = 0.220*X*_1_ + 0.274*X*_2_	2.951^**^	0.003
		3.679^***^	0.000
Step 3	*Y* = 0.115*X*_1_ + 0.121*X*_2_ + 0.249*M*	1.115	0.121
		1.617	0.107
		4.445^***^	0.000

*p < 0.05; **p < 0.01; ***p < 0.001.

## Discussion

This study explored the association between physical activity performed at home and overall well-being, while also examining the mediating influence of self-esteem. The results demonstrated that home-based physical activity positively predicted general well-being. Furthermore, self-esteem acted as a fully mediating factor in the relationship between medium and large amounts of home-based physical activity and overall well-being among university students, effectively serving as the pathway through which home-based physical activity affected general well-being. These main findings are discussed as follows.

### Relationship between home-based physical activity and general well-being

This study has revealed a positive correlation between home-based physical activity and general well-being, thereby supporting H1. These findings are in line with previous works demonstrating the relationship between physical activity and general well-being (Ibarzábal, 2017). Home-based physical activity thus plays a crucial role in promoting general well-being among individuals (Lesser and Nienhuis, 2020), leading to its recognition as an important health promotion behavior (Al-Ghafri et al., 2020).

Physical activity has some benefits, including the release of accumulated negative emotions (Chen et al., 2020), and is therefore crucial to maintain good mental health (Chekroud et al., 2018). Engaging in physical activity can provide effective relief from psychological pressure, leading to an overall improvement in general well-being (Ibarzábal, 2017). In light of the current pandemic, it is essential to ensure the maintenance of an adequate amount of physical activity among university students as it can significantly improve general well-being.

### Relationship between home-based physical activity and self-esteem

The present study revealed that engaging in home-based physical activity was positively correlated with self-esteem, thus corroborating H1. Additionally, it was verified that home-based physical activity had a significant impact on the level of self-esteem, which is in line with previous findings pertaining to the association between physical activity and self-esteem (Dale et al., 2019).

The conventional physical activity and self-esteem model posits that physical activity can substantially enhance an individual’s self-esteem (Lubans et al., 2016). A small-to-medium amount of physical activity has been evidenced to be positively related to higher self-esteem levels (Dale et al., 2019). Therefore, it can be concluded that home-based physical activity is an effective way to enhance self-esteem among university students during the COVID-19 pandemic.

### Mediating effect of self-esteem on the relationship between home-based physical activity and general well-being

It has been revealed that self-esteem fully mediated the relationship between home-based physical activity and general well-being in university students. This finding supports the hypothesis that home-based physical activity significantly predicts general well-being through the mediation of self-esteem.

Prior studies have also identified a link between home-based physical activity and self-esteem as well as the importance of physical activity in enhancing self-esteem (Hubbs et al., 2012; Kayani et al., 2018, Sani et al., 2016). Individuals with low levels of self-esteem struggle to effectively cope with challenges, hindering their general well-being (Leist and Müller, 2013). Conversely, individuals with higher self-esteem tend to have greater aspirations, resilience in the face of failure, and a lower likelihood of experiencing self-doubt or feelings of incompetence. As such, those with higher self-esteem are more likely to maintain a higher level of general well-being, even in the face of stress, trauma, and misfortune (Baumeister et al., 2003). Echoed by those findings, this study found that students with medium and large amounts of physical activity tend to develop a greater level of self-esteem, further contributing to their general well-being.

This study has several limitations. First, participants were drawn solely from two universities, resulting in a high degree of homogeneity; the internal validity of the study was high, which limits the external validity of the study. Second, the participants in this study were only limited to Chinese university students. It would be valuable to expand this study to include participants from diverse countries and populations. Third, in its inclusion criteria, our study investigators did not exclude participants with mental illness because they did not evaluate participants using mental health questionnaires, and/or other measures. This means that people suffering from various degrees of mental ill-health could have participated in our study, which may confound the results. Finally, while this study explored only one mediating variable that impacts the relationship between home-based physical activity and general well-being in university students, other relevant mediating variables remain unexplored. Further research should identify and study these variables, enriching relevant theoretical models in the process.

## Conclusion

In summary, the results of this study provide support for two hypotheses. Firstly, it was found that home-based physical activity demonstrated a positive correlation with the general well-being of university students during the COVID-19 pandemic. Secondly, it was observed that self-esteem acted as a mediator in the relationship between home-based physical activity and general well-being. These findings underline the significance of engaging in home-based physical activity to enhance the general well-being of university students during the pandemic. Students studying at universities are especially susceptible to the detrimental impacts of COVID-19-induced isolation. Thus, it is essential for their families to provide them with access to adequate sports facilities to ensure that they can engage in physical activities. Such activities play a significant role in maintaining both their physical and mental health, and can also help them develop well-rounded personalities, promoting their overall well-being.

## Data Availability

The original data are available on reasonable request from the corresponding author.
